# Analytical validation of the LungLB test: a 4-color fluorescence *in-situ* hybridization assay for the evaluation of indeterminate pulmonary nodules

**DOI:** 10.1186/s12890-024-03280-7

**Published:** 2024-09-27

**Authors:** Michelle L. Lutman, Daniel Gramajo-Leventon, Shahram Tahvilian, Lara Baden, Courtney L. Gilbert, Michael Trejo, Eric Vail, Michael J. Donovan, Benjamin A. Katchman, Paul C. Pagano

**Affiliations:** 1 LungLife AI, Inc., 2545 W. Hillcrest Drive, Suite 140, Thousand Oaks, CA 91320 USA; 2 Department of Pathology, Cedars Sinai Medical Center, Los Angeles, CA USA; 3 Department of Pathology, Icahn School of Medicine at Mount Sinai, New York, NY USA

**Keywords:** Indeterminate lung nodule, LungLB, Analytical validation, Circulating Genetically Abnormal Cell, CGAC

## Abstract

**Background:**

Evaluation of indeterminate pulmonary nodules (IPNs) often creates a diagnostic conundrum which may delay the early detection of lung cancer. Rare circulating genetically abnormal cells (CGAC) have previously demonstrated utility as a biomarker for discriminating benign from malignant small IPNs in the LungLB assay. CGAC are identified using a unique 4-color fluorescence *in-situ* hybridization (FISH) assay and are thought to reflect early cell-based events in lung cancer pathogenesis and the anti-tumor immune response. LungLB is a prognostic tool that combines the CGAC biomarker and clinical features to aid in IPN evaluation by improving the stratification of patient risk of malignancy.

**Methods:**

Herein we describe the analytical performance of the LungLB blood test. Analytical validation was performed according to Clinical and Laboratory Standards Institute (CLSI) guidelines with adaptations for rare cell-based assays. Multiple operators, reagent lots, and assay runs were tested to examine accuracy, precision, reproducibility, and interfering factors.

**Results:**

The FISH probes used in the LungLB assay demonstrate 100% sensitivity and specificity for their intended chromosomal loci (3q29, 3p22.1, 10q22.3 and 10cen). LungLB demonstrates analytical sensitivity of 10 CGAC per 10,000 lymphocytes analyzed, 100% analytical specificity, and high linearity (*R*^2^ = 0.9971). Within run measurements across 100 samples demonstrated 96% reproducibility. Interfering factors normally found in blood (lipemia, biotin) and exposure to adverse temperatures (-20ºC or 37ºC) did not interfere with results. Sample stability was validated to 96 hours.

**Conclusion:**

The analytical performance of LungLB in this validation study successfully demonstrates it is robust and suitable for everyday clinical use.

## Background

The majority of lung cancers are detected at an advanced stage, which is the reason why it is the leading cause of cancer-related mortality with over 120,000 deaths in the United States per year [1]. Computed tomography (CT) scan can detect lung cancer early when it is curable, however, the number of IPNs identified each year following CT scan exceeds 1.5 million and their management is challenging for an already burdened healthcare system [2]. Guidelines for nodule management are well-aligned for patients harboring nodules with high or low pretest risk of malignancy. However, intermediate-risk IPNs are a particular diagnostic challenge, and guidelines are mixed on when to recommend surveillance CT imaging, Positron Emission Tomography (PET) scan, or non-surgical biopsy in this population [3]. Non- and minimally invasive biomarkers are needed to help re-stratify intermediate-risk lesions into either 1) high-risk lesions that should be worked up quickly to avoid delays in diagnosis and treatment of potentially aggressive lung cancers, vs 2) low-risk lesions where less invasive monitoring procedures may be considered.

Using blood for early cancer diagnostics is a promising approach given the specimen can be obtained inexpensively and often less invasively than tissue biopsy, and is able to provide information more rapidly than surveillance-based imaging. Blood-based biomarkers for cancer detection have attracted significant research interest, especially in lung cancer where the biopsy procedure is invasive and not without potential complications [4]. Whole blood is a complex mixture that includes plasma and cell-based components, each of which contain unique biomarkers that are often complementary [5]. Plasma contains circulating cell free DNA (cfDNA and ctDNA from normal and tumor tissues, respectively), exosomal RNA, and various proteinaceous components. The cellular compartment contains erythrocytes, leukocytes, and tumor-derived cells which includes circulating tumor cells (CTC).

Emerging technologies for early detection of lung cancer focus on plasma biomarkers (cfDNA/ctDNA, RNA, or proteins) due to their relative ease of isolation and processing [6, 7] while cell-based biomarkers have largely been underutilized. However, there is a wealth of information in cells that can inform disease states and may better leverage natural disease processes that are active early in pathogenesis [8]. Previous studies demonstrated that CTCs can be identified in patients diagnosed with stage I lung cancer [9, 10]. Recently, CGAC have been described by our group and others to be predictive of cancer in patients presenting with IPNs [11–13], which is the basis of the LungLB test described herein. LungLB has demonstrated clinical validity using specimens from patients harboring indeterminate lung nodules [11], but analytical validity has not yet been reported.

Novel diagnostic tests must undergo rigorous analytical testing prior to clinical use to demonstrate accuracy and robustness in routine laboratory settings. This is especially true of assays based on rare cells, where generally accepted standards are not available. The CLSI is widely used to develop consensus-based laboratory standards that are accepted worldwide in order to improve patient care. The US FDA recognizes over 100 CLSI consensus standards, including those for method evaluation incorporated into this analytical validation [14]. Herein, we report on analytic sensitivity, specificity, accuracy, precision, linearity, and interference testing for LungLB.

## Methods

### Specimen collection, contrived samples, processing, and the LungLB assay

All samples were collected under an Institutional Review Board approved protocol (Advarra Pro00059106) and all volunteers were consented prior to blood draw. Samples used for this analytical validation were similar to clinical samples in terms of sample type, blood collection tube, collection method, quantity, and experimental process. Peripheral blood was collected from healthy adult volunteers (normal healthy donors, NHD) by standard venipuncture by a trained phlebotomist, as would be done in the clinical setting. Blood was drawn into a blood collection tube containing preservative (Streck, Omaha NE). Samples were stored for at least 24 h to simulate overnight shipping conditions utilized by ordering physicians.

Contrived samples were created by spiking A549 cells (ATCC: CCL-185) into blood from NHDs. A549 cells were used because they are a lung adenocarcinoma cell line and demonstrate copy number variation consistent with the previously described definition of a CGAC, which is a cell that has a gain in at least two of four FISH probes [11], compared to a normal cell which is diploid for all four probes. The FISH probes used in LungLB have demonstrated similar CNV patterns and clinical performance in detecting CGACs in patients with lung adenocarcinoma, squamous cell carcinoma, and small cell/carcinoid tumors [11]. A549 and blood cells were counted using a BD Accuri C6 flow cytometer (BD San Jose, CA). Samples were spiked at 5 (low), 10 (medium), and 20 (high) A549 cells per 10,000 lymphocytes. Non-spiked blood was used as the negative sample.

**Fig. 1:**
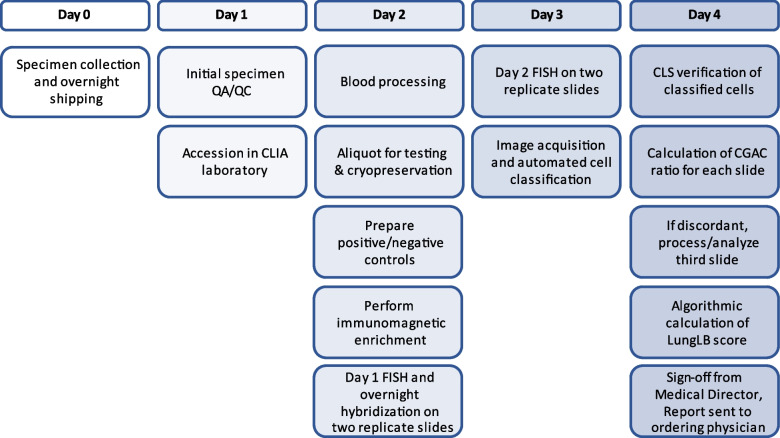
LungLB test process flow diagram from blood draw to report

All samples were accessioned and processed in LungLife’s CLIA-certified laboratory by trained and competent personnel according to standard operating procedures (SOPs) as previously described [11] and as outlined in Fig. 1***.*** Studies incorporating CLSI standards were designed to measure probe sensitivity and specificity, limit of blank (LoB), linearity, precision, accuracy and interfering factors using NHD blood with and without spiked A549 cells (Table 1). Briefly, blood was centrifuged at 1000xg for 10 min with the brake off. Plasma was collected and stored at -80ºC and erythrocytes were lysed using an ammonium chloride-based lysis solution. Recovered leukocytes were counted using flow cytometry. All parts of the LungLB assay, including immunomagnetic isolation of CGAC, FISH, and image acquisition/analysis were performed as previously described [11].

**Table 1:** Summary of analytical validation studies of LungLB

Performance characteristic	Definition	CLSI Standard	Sample description	Measured analytic parameter
**Probe sensitivity, specificity**	FISH on metaphase chromosomes	MM07-A2	30 metaphase cells across 6 slides from male NHDs	Percent sensitivity and specificity (probe)
**Limit of blank**	Highest measurement likely observed in blank samples	EP17-A2	32 NHDs and 17 confirmed benign samples	Threshold at target percent specificity
**Linearity**	Proportionality of results to concentration of analyte	EP05-A3	Sextuplicate runs of 0, 5, 10, 20 spiked cell blood samples	Linear regression with R^2^ value
**Precision**	Intra-assay, inter-assay and inter-operator reproducibility	EP05-A3	100 slides across 3 reagent lots, 4 operators, and 5 days	Percent concordance across all slides (qualitative)
**Accuracy**	Degree of agreement between result and spiked value	EP24-A2	Sextuplicate runs of 0, 5, 10, 20 spiked cell blood samples	Sensitivity (CGAC ratio) and percent specificity (assay)
**Interfering factors**	Substances that may alter the result of an assay	EP07 EP25 EP37 C56-A	Duplicate slides, multiple timepoints testing temperature, over-fixation, lipemia, biotin	Concordance with baseline result (qualitative)

*FISH* Fluorescence in-situ hybridization, *CLSI* Clinical and Laboratory Standards Institute, *NHD* Normal Healthy Donor

### FISH probe sensitivity and specificity

Probe hybridization sensitivity and specificity were assessed in accordance with CLSI MM07-A2 by processing metaphase cells using the LungLB assay, which is performed anytime a new lot of FISH probes is made [15]. Blood was collected in sodium heparin vacutainer tubes from male healthy donors in order to assess binding to all possible chromosome locations. Blood cells were incubated for 72 h in RPMI-1640 with mitogen to activate cell division. Cultured blood cells were treated with colcemid to induce metaphase arrest and a hypotonic potassium chloride solution according to standard practices. Treated cells were suspended in Carnoy’s fixative and dropped on to clean glass microscope slides and air dried. Following G-banding, cells were imaged and Chromosomes 3 and 10 were labeled using CytoVision (Leica Biosystems, Deer Park, IL) software. The same FISH procedure described previously [11] was then performed and slides were re-imaged and analyzed using the CytoVision software. For autosomal targets, 100 metaphase cells (with unbroken and non-overlapping nuclei) must be analyzed per CLSI guidelines, and FISH probe sensitivity and specificity must equal or exceed 95% and 98%, respectively, for suitability for FISH testing. Analysis can be terminated at 20 cells if the assessment has been performed on metaphase cells and the sensitivity and specificity are both 100% at that point in the assessment. Sensitivity is calculated by dividing the total number of signals at the intended chromosomal region by the total number of intended targets in the cells examined. Probe specificity is calculated by dividing the total number of signals at the intended chromosomal region by the total number of signals observed over all chromosomal regions.

### Analytical specificity (limit of blank)

The LungLB test is a qualitative assay that provides an “Increased Risk” (positive) or “Decreased Risk” (negative) result using a normalized CGAC ratio, which is a function of the CGACs recovered divided by total cells imaged. The experimental design consisted of replicate measurements from thirty-two NHDs and seventeen confirmed benign lung nodule samples across six non-consecutive days using three unique reagent lots. This accounts for a total of 98 samples using 196 slides, fitting guidelines in CLSI EP17-A2 [16]. The final LoB calculation uses the results of the normalized CGAC ratios for all runs, and was applied to determine the threshold cutoff of the LungLB test using the parametric option for data analysis:

$$LoB = {M}_{B} + {c}_{p}{SD}_{B}$$

where *M*_*B*_ is the mean and *SD*_*B*_ is the standard deviation of the blank samples, respectively, and *c*_*p*_ is the multiplier (0.05 for 70th percentile, based on the intended use specificity/proportion of false positives (α) less than 30% [11, 16]. Limit of Detection (LoD) is not reported as part of this analytical validation because the microscope is capable of single-cell resolution.

### Linearity, reproducibility, and analytical accuracy

Contrived samples at each spike condition (0, 5, 10 and 20 A549 per 10,000 lymphocytes) were run in triplicate by multiple operators (*n* = 2) for a total of 24 samples, and linear regression analysis was used to determine linearity.

To determine reproducability replicate measurements on twenty clinical samples from patients with indeterminate lung nodules, equally distributed across multiple unique reagent lots (*n* = 3) and operators (*n* = 4), were performed. Each sample was run in duplicate across five non-consecutive runs, resulting in 200 slides and 100 total individual test results. This aligns with a 3 × 5 × 5 × 2 alternative design described in CLSI EP05-A3 [17]. If the two replicate slides used for a sample were discordant, a third slide was used for the final result of the sample, as is done clinically [11].

The analytical sensitivity, specificity, and accuracy were determined according to CLSI EP24-A2 [18]. Six contrived samples were processed per spike condition (0, 5, 10 and 20 A549 per 10,000 lymphocytes) for a total of 24 samples. Native CGAC from NHD blood samples were excluded and only spiked A549 cells were counted. Analytic sensitivity is defined as the lowest spike condition that will return a positive result 95% of the time or more in the LungLB assay. Analytic specificity is calculated by dividing the number of negative results by the sum of negatives and positive results from LungLB tests on non-spiked NHD blood. Analytic accuracy is reported as the sum of the correctly classified samples divided by the total samples run at the spike conditions determined for analytic sensitivity and used for analytic specificity.

### Interfering factors

Studies were performed to evaluate factors influencing sample stability, including transport temperature (2-8ºC, 37ºC, and freeze/thaw cycle) and fixation time (24 h up to 7 days), according to CLSI EP25-A [19] or the effects of endogenous substances (biotin and lipemia) and blood tube preservative (over-fixation) in accordance with CLSI EP07, EP37 and C56-A [20–22]. Storage temperatures were verified and monitored using calibrated digital monitoring systems (Monnit, Salt Lake City, UT) or calibrated NIST-traceable thermometers (Fisher Scientific). Lipemia (10 mg/mL Intralipid) and Biotin (3,500 ng/mL) were tested as potential interfering factors, the latter because the LungLB test incorporates streptavidin conjugated beads for immunomagnetic enrichment of CGAC. LungLB was performed on non-spiked or spiked (20 A549 / 10,000 lymphocytes) NHD blood in duplicate for each condition on two different days to understand the effects of interfering factors on test performance. Single positive and negative control samples are performed with each run for each condition. The interfering factor was introduced to the blood sample prior to processing, thus the evaluation of interference was designed to determine how the entire test process post-blood collection could be affected.

### Data analysis

The Mann–Whitney test was used to compare average CGAC counts between NHD and patients with benign lung nodules. All graphs were created and linear regression analysis was performed using GraphPad Prism software (Boston, MA).

## Results

### FISH probe sensitivity and specificity

**Fig. 2:**
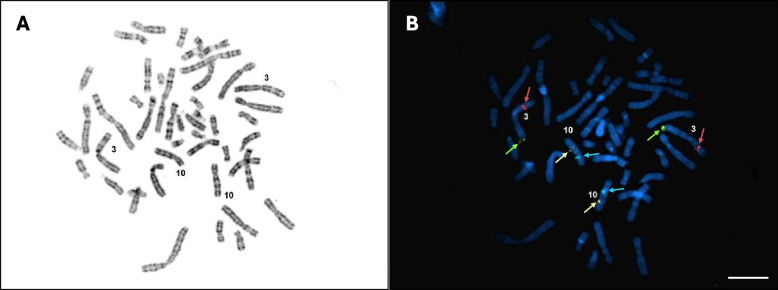
Probe sensitivity and specificity using metaphase chromosomes. **a** Representative image taken from G-banded metaphase slide. Chromosomes 3 and 10 are labeled for reference. **b** Staining with the LungLB FISH probe mix is shown for Chromosomes 3q29 (Green), 3p22.1 (Red), 10q22.3 (Gold), and 10CEN (Aqua). Color-coded arrows indicate LungLB FISH probe signals and scale bar is 5 µm

LungLB is a qualitative 4-color FISH assay utilizing four unique probes to target chromosome locations 3q29 (Green), 3p22.1 (Red), 10q22.3 (Gold), and 10cen (Aqua), allowing for visual analysis of copy number variation (CNV) using wide-field fluorescence microscopy. These genetic loci were selected as they demonstrated robust CNV in individuals with primary lung adenocarcinoma and squamous cell carcinoma as determined by array CGH [23]. Probe hybridization sensitivity and specificity were assessed in accordance with CLSI MM07-A2 [15]. Thirty metaphase spreads across slides from six unique known normal XY blood samples were procured and FISH was performed on these slides. (Fig. 2). All four color probes were identified on each analyzed metaphase cell with hybridization to the correct region, therefore probe sensitivity and specificity were 100%.

### Analytical specificity (Limit of Blank, LoB)

**Fig. 3:**
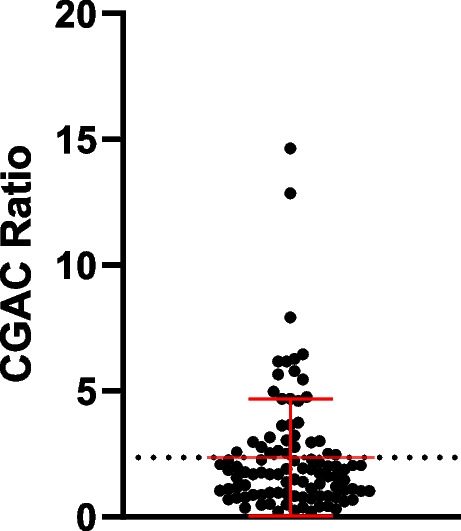
Limit of Blank. LungLB Limit of Blank (LoB) was determined from 98 individual sample runs of blood from NHD or patients with confirmed benign lung disease. The line represents a LoB threshold ratio of 2.47. Error bars are mean ± SD

To understand the highest measurement result that is likely to be observed for a blank sample, we performed a LoB study using NHD blood and benign lung disease (from biopsy-confirmed indeterminate lung nodules) patient samples according to CLSI EP17-A2 [16]. The LoB threshold for CGAC was determined to be 2.47 (Fig. 3) using 98 determinations (64 NHD and 34 confirmed benign slides). There was no statistically significant difference in the mean CGAC ratio of NHD and benign samples (Mann–Whitney, *P* = 0.3611).

### Linearity and analytical precision/reproducibility

**Fig. 4:**
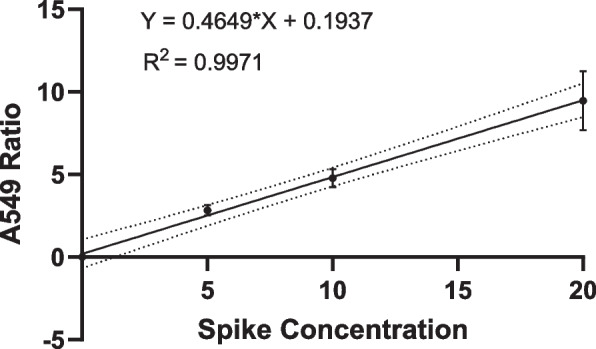
LungLB Linearity. LungLB Linearity was determined by processing contrived samples spiked at 5, 10, and 20 A549 / 10,000 lymphocytes. Each point is the mean normalized A549 counts across six independent runs per spike condition, and 95% confidence bands are displayed (dotted lines)

A series of experiments to understand assay precision and reproducibility were performed according to CLSI EP05-A3 [17]. To calculate assay linearity, a simple linear regression was performed using six replicate samples at four spike concentrations. The assay demonstrated a high degree of linearity across the expected range of CGAC recovery with *R*^2^ = 0.9971 (Fig. 4).

Slide-to-slide (intra-day) and day-to-day (inter-day) precision results were also evaluated using blood from patients with indeterminate lung nodules. Normalized CGAC ratios were calculated for each sample on each run/day and each normalized value was classified as Increased Risk (at or above cutoff) or Decreased Risk (below cutoff). Table 2 summarizes the results for individual slides (2 slides per patient), which collectively demonstrated 96% reproducibility across all 100 samples.

**Table 2:** Summary of results for within run precision and reproducibility

Within run summary table			
Run number	**Number of slides**	^**a**^**Discordant slides**	**Percent precision**
**Run 1**	40	1	97.5%
**Run 2**	40	2	95.0%
**Run 3**	40	0	100.0%
**Run 4**	40	3	92.5%
**Run 5**	40	1	97.5%
**Summary**	(100) final results	(4) Discordant Results	96.0%

^a^Discordant Slides refers to a qualitative difference in the single slide result of replicate slides run on a single day. The majority of discordant slides represent a single-cell difference in CGAC count

### Analytical accuracy

**Fig. 5:**
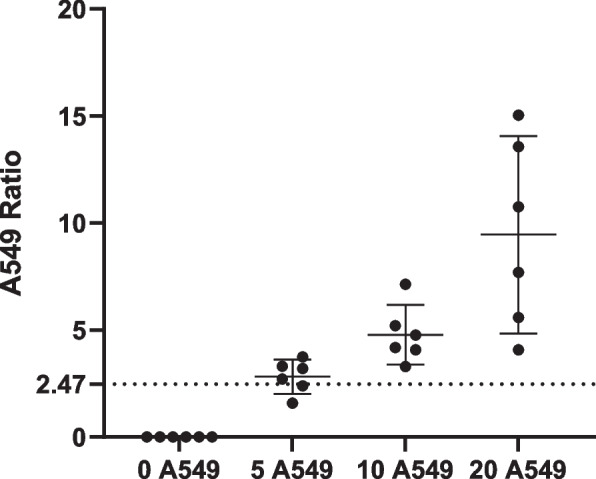
Samples were processed in triplicate per spike concentration by two unique operators. The dotted line at 2.47 represents the threshold identified from LoB experiments. Dots are individual normalized A549 ratios and error bars are mean ± 95% CI

The analytical sensitivity, specificity, and accuracy were determined according to CLSI EP24-A2 [18]. The individual and average normalized A549 ratio across six replicate samples processed per spike condition are demonstrated (Fig. 5). All high and medium range spiked samples produced positive results above the established threshold to reach 100% (12/12) accuracy. Low range spiked samples produced four positive results and two negative results for 66.67% accuracy at the LoB threshold. Therefore, the analytical sensitivity for LungLB is established at 10 A549 / 10,000 lymphocytes (Fig. 5). Notably, the two negative results at the low spike were due to a difference of a single A549 cell. To determine analytic specificity, 32 NHD were tested, all producing a negative result (no A549 cells found), for 100% analytical specificity.

The total analytical accuracy was calculated using the analytical specificity and sensitivity results described above. All 12 samples spiked at or above the analytic sensitivity (10 A549 / 10,000 lymphocytes) produced a positive test result, and all 32 NHD samples produced a negative test result. As such all 44 results were concordant for a 100% total analytical accuracy.

### Interfering factors

Studies were performed to evaluate factors influencing sample stability, including transport temperature and fixation time, according to CLSI EP25-A [19]. Additionally, the effects of endogenous substances (biotin and lipemia) and blood tube preservative (over-fixation) on assay performance were tested in accordance with CLSI EP07, EP37 and C56-A [20–22]. Baseline results were established using the ideal sample conditions: full (4—5 mL) blood draw and room temperature (16 °C—26 °C) storage for 24 h and 7 days (Table 3).

**Table 3:** Interference – temperature and stability

	***Sample type***	***24 h result***	***7 day result***
***RT (baseline)***	** + *Control***	Positive	Positive
	***- Control***	Negative	Negative
	***High C95***	Increased Risk	Increased Risk
	***Low C5***	Decreased Risk	Decreased Risk
***2-8ºC***	** + *Control***	Positive	Positive
	***- Control***	Negative	Negative
	***High C95***	Increased Risk	Increased Risk
	***Low C5***	Decreased Risk	Decreased Risk
***37ºC***	** + *Control***	Positive	Positive
	***- Control***	Negative	Negative
	***High C95***	Increased Risk	Increased Risk
	***Low C5***	Decreased Risk	Decreased Risk
***-20ºC***	** + *Control***	Positive	n/a
	***- Control***	Negative	n/a
	***High C95***	Increased Risk	n/a
	***Low C5***	Decreased Risk	n/a

LungLB was performed on contrived samples spiked with A549 cells stored at different conditions to understand the effects of temperature on test performance. “RT” is Room Temperature, “ + Control” is Positive Control, “- Control” is Negative Control, “High C95” is the concentration that produces 95% positive results, or 20 A549 cells/10,000 lymphocytes spike, “Low C5” is the concentration that produces 5% positive results, which is non-spiked. Two slides were run for each condition tested and both yielded concordant qualitative results. One time point was tested for -20ºC samples to mimic a single freeze thaw cycle

For temperature-based interference testing, storage at 50ºC resulted in coagulation within 24 h, and storage at 40ºC resulted in quality control (QC) rejection due to insufficient cells recovered. Samples stored at 37 °C for 24 h and 7 days passed all quality assurance steps and yielded concordant results. This demonstrates that assay quality assurance requirements will detect preanalytical temperatures outside validated stability ranges and that the blood specimen is stable for up to 7 days at room temperature and up to 37 °C (Table 3).

Shipping could temporarily expose specimens to freezing conditions (freeze thaw cycle). To simulate this, samples were placed in a -20 °C freezer for 24 h and were then thawed to room temperature before processing. The freeze thaw cycle induced total hemolysis of all samples and left only a small pellet of nucleated cells after the first centrifugation step of the procedure. The hemolyzed samples were processed fully and analyzed, and the samples passed all quality metrics used for the LungLB test and produced appropriate results, demonstrating that freezing does not adversely affect the LungLB test results. Excessive cold without freezing was also tested by storing samples at 2-8ºC for the same time period with no impact on reported result (Table 3).

The blood collection tube contains an aldehyde-releasing stabilization solution that cross-links biomolecules. Over-fixation of the sample may be possible with low blood-to-stabilization solution ratio following collection. Over-fixation was modeled by drawing approximately 2 mL of blood into the collection tube designed for 5 mL of blood. While these samples passed at the 24-h timepoint, the short draw yielded too-few cells at the 7-day timepoint and therefore failed quality assurance (< 10,000 cells on the slides). The experiment was then repeated using a 96-h timepoint with slides achieving concordant results (Table 4). Therefore, short draw (2—2.5 mL) samples must be processed prior to 96 h from the time of draw. Samples with less than 2 mL of blood drawn typically do not have sufficient leukocytes for LungLB and will therefore be rejected.

**Table 4:** Interference – chemical interferents and stability

	*Sample type*	*24 h result*	*96 h result*	*7 day result*
***Over fixation***	** + *Control***	Positive	Positive	Positive
	***- Control***	Negative	Negative	Negative
	***High C95***	Increased Risk	Increased Risk	Failed QC
	***Low C5***	Decreased Risk	Decreased Risk	Failed QC
***Lipemia***	** + *Control***	Positive	Positive	Positive
	***- Control***	Negative	Negative	Negative
	***High C95***	Increased Risk	Increased Risk	Failed QC
	***Low C5***	Decreased Risk	Decreased Risk	Decreased Risk
***Biotin***	** + *Control***	Positive	Positive	Positive
	***- Control***	Negative	Negative	Negative
	***High C95***	Increased Risk	Increased Risk	Failed QC
	***Low C5***	Decreased Risk	Decreased Risk	Decreased Risk

LungLB was performed on contrived samples spiked with A549 cells stored at different conditions to understand the effects of various chemical interferents on test performance. “ + Control” is Positive Control, “- Control” is Negative Control, “High C95” are samples spiked at 20 A549 cells/10,000 lymphocytes, which are expected to yield an “Increased Risk” result, and “Low C5” are non-spiked samples, which are expected to yield “Decreased Risk” result. Two slides were run for each condition tested and both yielded concordant qualitative results

To determine if lipemia interferes with the LungLB test, 10 mg/mL of Intralipid was spiked into blood and incubated with the samples at room temperature for 24 h and 7 days. One of the slides for the 7-day samples yielded less than 10,000 cells scanned and did not pass quality assurance. Lipemia spiked samples were then tested at a 96-h timepoint with concordant results (Table 4).

CGAC isolation occurs using streptavidin magnetic beads and endogenous biotin may affect assay performance. To align with FDA guidance and CLSI EP37, biotin was added to NHD blood to a final concentration of 3,500 ng/mL, roughly three times the maximum expected clinical concentration, and was incubated at room temperature for 24 h and 7 days. One of the slides for the 7-day samples yielded less than 10,000 cells scanned and did not pass quality assurance. Biotin spiked samples were then tested at 96-h timepoint with concordant results (Table 4). This is consistent with the short-draw and lipemia results, and as such sample stability can only be validated to 96 h post blood draw.

## Discussion

It is critical for new laboratory tests to be both clinically and analytically validated prior to clinical use. Any anticipated sources of variability and interference during the preanalytical and analytical phases of testing must be assayed in a controlled setting and in accordance with recognized CLSI standards to understand the impact on test performance. This helps provide the framework for sample acceptance/rejection criteria and helps to ensure a quality product. This report details the results of probe sensitivity/specificity, analytical sensitivity, specificity, precision, and the impact of interfering substances on LungLB test performance in a validation aligned with CLSI standards and supports the previously described clinical validity.

Sample stability is an important metric to understand when assessing reliability of a test result. While 7-day stability is feasible under ideal and temperature-challenged conditions (Table 3), it seems certain interfering factors may influence sample stability at 7 days (Table 4). Therefore, sample stability can only be validated to 96 h post blood draw. While our experience is that blood samples can be shipped from anywhere in the United States and most arrive at our laboratory within 24 h, future studies involving stability extension beyond 96 h are being explored.

Limit of Detection (LoD) is a commonly used endpoint in analytical validation assays for clinical chemistry. However, for rare-cell based assays employing FISH, the LoD is one cell because the microscope is capable of single-cell resolution. This is reflected in CLSI MM07, where “LoD cannot be used for FISH because there is no such thing as a “blank” sample and because, without better measurement technology, it is usually not possible to produce mixtures of cells in which the frequency of abnormal cells is close to the LoD” [15]. Therefore, LoD is not reported as part of this analytical validation.

Spiking a small number of cells into blood with accuracy and/or precision is challenging and results in significant variation [24]. One cell difference at a five-cell spike results in 20% variance. 67% analytic sensitivity at the 5 A549 spike condition is likely underestimated: average recovery is reported to be 50% at each spike condition (Fig. 5), which is an artifact of contrived samples not applicable to clinical specimens. Had one additional cell been recovered in the two false negative runs, sensitivity would have been higher at this spike condition. That said, even with low recovery LungLB showed excellent assay linearity across a range of expected clinical concentrations (*R*^2^ = 0.9971).

Indeterminate lung nodules can create a diagnostic challenge for physicians. While existing tools such as PET and nodule calculators are used to help physicians understand the likelihood a nodule is malignant, they are unreliable for smaller lesions or in areas of endemic granulomatous disease [25, 26]. LungLB is intended to be used as an aid in the evaluation of indeterminate lung nodules, and has demonstrated better performance than both PET and the Mayo nodule calculator, especially for smaller lesions, in patients undergoing percutaneous biopsy [11, 27]. This supports the utility of LungLB, which may complement existing methodologies and add to the physician’s armamentarium to detect lung cancer early. Additional studies to further support clinical validity are underway.

## Conclusions

Based on the data in this analytical validation, LungLB was demonstrated to be robust to common variations in the laboratory environment.

## Data Availability

The datasets generated during the current study are not publicly available due to concerns regarding participant confidentiality and proprietary information but are available upon reasonable request from the corresponding author.

## Abbreviations

IPN: Indeterminate pulmonary nodule
CGAC: Circulating genetically abnormal cell
FISH: Fluorescence *in-situ* hybridization
CLSI: Clinical and Laboratory Standards Institute
CT: Computed tomography
cfDNA: Circulating cell-free DNA
ctDNA: Circulating tumor DNA
CTC: Circulating tumor cell
NHD: Normal healthy donor
SOP: Standard Operating Procedure
CNV: Copy Number Variation
QA: Quality Assurance
QC: Quality Control
LoB: Limit of Blank
LoD: Limit of Detection
SD: Standard Deviation
CI: Confidence Interval
